# Evidence for Threshold-like Dynamics in *Aedes* Mosquito Populations Under Sustained Mass Trapping on Tropical Islands

**DOI:** 10.3390/insects17050472

**Published:** 2026-05-02

**Authors:** Maximilian Epple, Andreas Rose, Martin Geier, Bart G. J. Knols

**Affiliations:** 1 Biogents AG, An der Irler Höhe 3a, 93055 Regensburg, Germany; maximilian.epple@biogents.com (M.E.); andreas.rose@biogents.com (A.R.); martin.geier@biogents.com (M.G.); 2 K&S Holding BV, Kalkestraat 20, 6669 CP Dodewaard, The Netherlands; 3 Ifakara Health Institute, Ifakara P.O. Box 53, Tanzania

**Keywords:** *Aedes*, mosquito, mass trapping, model, trap density, population dynamics, islands, vector control, tropics, arbovirus

## Abstract

Mass trapping of adult mosquitoes is increasingly promoted as an environmentally friendly alternative to insecticide-based vector control, yet quantitative evidence for its long-term population-level effects remains limited. We analyzed adult *Aedes* mosquito Biogents trap data from four tropical islands (three in the Maldives, one in the Philippines) where mass trapping was implemented at different trap densities. Using equilibrium-constrained population models, we describe how adult *Aedes* populations differ across trap densities, with outcomes ranging from partial suppression to near-zero levels at higher trap densities. At low to intermediate densities (4–6 traps·ha^−1^), populations stabilized at non-zero equilibrium levels, whereas operational elimination was consistently observed at densities ≥ 10 traps ha^−1^. A descriptive curve is shown to illustrate the decline in equilibrium abundance with increasing trap density, while a conceptual sigmoid model is used to illustrate how a transition in the recruitment–removal balance may occur under theoretical conditions. Limited larval source management was implemented on two islands, but elimination was also observed in the absence of larval interventions, indicating that sustained adult removal appears to have been the dominant driver of suppression. These findings indicate that mass trapping, when deployed at sufficiently high densities, is associated with rapid declines to near-zero population levels and may serve as an effective component of integrated vector management, particularly in geographically bounded settings or as a rapid-response intervention during outbreaks of arboviral diseases.

## Simple Summary

*Aedes* mosquitoes spread diseases such as dengue, Zika, and chikungunya, and rapid mosquito control is especially important during outbreaks. One increasingly used method is mass trapping, where many traps are deployed to remove adult mosquitoes, but its long-term effectiveness is not well documented. We analyzed several years of mosquito trapping data from four tropical islands with different Biogents trap densities. At low to moderate trap densities (4–6 traps·ha^−1^), mosquito populations declined but stabilized at reduced levels. At higher densities (10–15 traps·ha^−1^), adult populations declined to near-zero levels and, in some cases, remained undetectable for extended periods. These findings suggest that sufficiently high trap densities can shift mass trapping from partial suppression to operational elimination in settings with restricted mosquito immigration.

## 1. Introduction

Mosquito-borne diseases remain among the most significant and rapidly evolving global public health challenges. Over the past two decades, the epidemiology of arboviral diseases like dengue, Zika, chikungunya, and yellow fever has changed markedly, with substantial increases in geographic range, incidence, and epidemic frequency. Dengue alone has increased more than eightfold since 2000, with an estimated 390 million infections annually, of which approximately 96 million are clinically apparent. Recent surveillance data indicate that reported cases alone have reached unprecedented levels, exceeding 14 million globally in 2024, highlighting both increasing transmission and improved detection [1,2,3]. Once largely confined to tropical regions, arboviruses are now established or emerging in subtropical and temperate areas, including southern Europe, the United States, and East Asia [4,5,6].

Multiple interacting drivers underpin this expansion. Intensification of global trade has facilitated the transport of mosquito vectors, particularly *Aedes aegypti* and *Ae. albopictus*, via shipping containers, used tires, and ornamental plants [7,8]. International travel has increased the frequency of virus importation into receptive regions, enabling local transmission where competent vectors are present [9]. Concurrently, climate change has altered temperature and precipitation patterns in ways that enhance mosquito survival, shorten viral extrinsic incubation periods, and expand seasonal transmission windows [10,11,12]. Rapid and often unplanned urbanization has further amplified risk by creating dense human populations with abundant artificial larval habitats and limited access to effective vector control [3,13].

Despite these changing dynamics, vector control strategies have remained heavily reliant on chemical insecticides for much of the past half century. Adulticiding (predominantly through fogging or misting with synthetic pyrethroid insecticides) has been a central component of national and municipal mosquito control programs worldwide. However, resistance to commonly used insecticides is now widespread in *Aedes* populations, with documented resistance to pyrethroids and organophosphates reported across Asia, Africa, the Americas, and the Pacific [14,15]. Resistance not only reduces immediate operational effectiveness but also undermines outbreak response capacity during epidemics. Larval source management (LSM), including larviciding with biological control agents (e.g., *Bacillus thuringiensis israelensis*) and insect growth regulators (IGRs) such as methoprene and pyriproxyfen, is promoted as a more environmentally sound alternative. To date, resistance to IGRs remains comparatively limited. While this rarely leads to operational failure, it underscores the importance of resistance management and diversified control strategies within integrated vector management frameworks.

Beyond resistance, the ecological and societal costs of chemical-based control are receiving growing scrutiny. Broad-spectrum insecticides can negatively affect non-target organisms, including pollinators, aquatic invertebrates, and other components of local biodiversity [16,17,18]. Public concern regarding environmental contamination, human exposure, and impacts on ecosystem services has contributed to declining acceptance of routine space spraying, particularly in environmentally sensitive settings such as islands, resorts, and protected areas. Together, these challenges have driven an urgent search for alternative, sustainable, and socially acceptable vector control strategies.

In response, several innovative approaches have been developed and deployed over the past two decades. These include the release of *Wolbachia*-infected mosquitoes to reduce vector competence for arboviruses, population suppression through sterile insect technique (SIT), and genetic engineering approaches such as gene drive systems [19,20,21,22]. While promising, these methods present their own operational, regulatory, and societal challenges. High implementation costs, logistical complexity, regulatory uncertainty, and public acceptance remain barriers to large-scale deployment in many settings. Moreover, some approaches require sustained releases or centralized production facilities, which may limit feasibility in remote or resource-constrained environments.

Against this backdrop, mass trapping has emerged as a potential complementary or alternative vector control strategy [23]. Unlike surveillance trapping, mass trapping aims to remove a substantial proportion of the adult mosquito population continuously, thereby reducing vector density, biting rates, and ultimately disease transmission. Advances in trap design, particularly CO_2_- and kairomone-baited traps targeting host-seeking females, have improved capture efficiency and species specificity. For *Aedes aegypti* and *Ae. albopictus*, Biogents traps (e.g., BG-Mosquitaire/BG-Sentinel) have demonstrated high attractiveness and sustained operational robustness under a wide range of field conditions, including large-scale control programs [24,25].

Empirical evidence supporting mass trapping as a control intervention has grown in recent years, particularly in geographically isolated or semi-closed systems. Island environments, in particular, provide valuable natural laboratories for evaluating population-level impacts due to limited immigration and well-defined boundaries. Field studies in Maldivian islands have shown that sustained deployment of adult traps can drive dramatic reductions in *Aedes albopictus* and *Culex quinquefasciatus* populations, in some cases approaching local elimination [26]. Similar outcomes have been reported from resort islands in the Philippines and other south-east Asian countries, where intensive trapping reduced adult mosquito abundance and nuisance biting over extended periods or even resulted in elimination of *Ae. aegypti* and *Culex quinquefasciatus* [27].

Despite these encouraging results, key questions remain unresolved. In particular, the relationship between trap density and long-term population outcomes has not been quantified across multiple operational settings. It remains unclear whether mass trapping exhibits linear responses to increasing trap density or whether threshold-like dynamics may arise under certain conditions, whereby removal exceeds local population growth (i.e., recruitment from immature stages within the island system, not immigration, which is considered negligible). While addressing this question would require replicated observations across a range of trap densities, comparative data from operational systems can provide initial insights into how different trapping intensities correspond to observed population outcomes. Clarifying these patterns is important for translating empirical observations into operational guidance.

Here, we combine long-term field data from multiple islands with a simple mechanistic population model that balances mosquito recruitment against trap-mediated adult removal; an approach that mirrors recruitment–removal frameworks widely used to analyze pheromone-based mass trapping, mating disruption, and sterile insect technique (SIT) programs [28,29,30]. Using comparative data from four islands differing in size, mosquito species present, ecology, and trap density, we estimate equilibrium mosquito abundance under sustained and continuous trapping and describe how population outcomes differ across these systems. The patterns observed across systems provide a descriptive basis for understanding how varying trapping intensities correspond to different long-term population levels, offering practical guidance for designing scalable and environmentally sustainable mosquito control programs on islands and other semi-contained settings.

## 2. Materials and Methods

### 2.1. Study Sites

Mass trapping operations were implemented on four tropical islands with sustained adult mosquito trapping programs: Puerco (Palawan, Philippines; 7.2 ha, starting in July 2022), Kunfunadhoo (Baa atoll, Maldives; 41.4 ha, starting in March 2019), Medhufaru (Noonu atoll, Maldives; 49.0 ha, starting in April 2020), and Dhipparufushi (Haa Dhaalu atoll, Maldives; 3.0 ha, starting in July 2021). All trapping operations continue to date. The Maldives islands are in the Central Indian Ocean region, Puerco island in the Western Pacific Ocean; all islands experience average daily temperatures of 28–32 °C and relative humidity typically exceeding 80%, allowing year-round mosquito breeding. Islands differed in size, vegetation structure, human occupancy, and trap density, but all were characterized by clearly defined boundaries and very limited mosquito immigration (Table 1). The islands have been described in more detail by Jahir et al. [26] and Knols et al. [27].

### 2.2. Mass Mosquito Trapping and Data Collection

Adult mosquitoes were collected using BG-Mosquitaire traps (Biogents AG, Regensburg, Germany; see Jahir et al. [26] for a full description of the trap system) baited with synthetic human odor lures and CO_2_ supplied via a yeast-sugar-water fermentation system, as described previously [26,27]. Traps were operated continuously (24 h·day^−1^) throughout the study periods. Trap densities varied among islands (Table 1) and were maintained as constant as possible during each deployment: approximately 10 traps·ha^−1^ on Puerco (n = 72), 6 traps·ha^−1^ on Kunfunadhoo (n = 250), 4.1 traps·ha^−1^ on Medhufaru (n = 200), and 15 traps·ha^−1^ on Dhipparufushi (n = 45).

Trap catches were collected at 3-day intervals. Adult *Aedes* mosquitoes were identified morphologically and enumerated. Mean catch per trap per day was calculated for each sampling date and used as a proxy for adult population abundance. This proxy is widely used as an operational index of adult mosquito abundance in vector surveillance and ecological studies, particularly when standardized trapping effort is maintained across time and space [24,31,32]. For all islands, *Aedes* and *Culex* mosquitoes were recorded; all analyses presented here focus exclusively on *Aedes*. For the analyses presented here, data were included from the first 300 days following initiation of trapping on each island. Start dates were: Kunfunadhoo (March 2019), Medhufaru (April 2020), Dhipparufushi (July 2021), and Puerco (July 2022).

### 2.3. Rainfall Data

Daily rainfall data were obtained from on-site meteorological stations or nearby weather stations (except for Dhipparufushi) and expressed as millimeters per day. Short-term fluctuations in adult abundance were expected to coincide with rainfall events. Similarly, rainfall would contribute to hatching of dormant eggs once these became flooded, resulting in increased densities.

### 2.4. Population Model and Parameterization

Adult *Aedes* population dynamics under sustained mass trapping were described using a deterministic recruitment–removal model that captures the balance between adult mosquito recruitment and trap-mediated adult mortality. The model assumes that, at the island scale, adult population dynamics can be approximated by a first-order process in which trapping imposes a continuous removal pressure on the standing adult population.

The model is expressed as follows:

*y*(*t*) = *y_∞_* + (*y*_0_ − *y_∞_*) *e*^−*rt*^

where *y*(*t*) represents adult mosquito abundance at time *t* (days since initiation of trapping), *y*_0_ is the baseline abundance at the start of trapping (day 0), *r* is the net removal rate (day^−1^), and *y*_∞_ is the long-term equilibrium abundance.

To facilitate comparison across islands with different absolute mosquito densities and trap numbers, abundance was normalized and expressed as a percentage of baseline abundance (*y*_0_ = 100%) for all analyses.

#### 2.4.1. Model Interpretation

The parameter *r* represents the net per capita rate at which adult mosquitoes are removed from the population, incorporating both trap-induced mortality and background adult mortality. Higher values of *r* indicate faster population decline following trap deployment. The equilibrium abundance *y*_∞_ reflects the balance between adult recruitment (emergence of new adults from immature stages) and adult removal. When *y*_∞_ approaches zero, adult removal exceeds recruitment, resulting in population collapse or near-elimination. Non-zero values of *y*_∞_ indicate partial suppression, with continued recruitment sustaining the population at a reduced level.

#### 2.4.2. Parameter Estimation

Model parameters *r* and *y*_∞_ were estimated separately for each island using nonlinear least-squares fitting to observed time series of mean adult *Aedes* catch per trap per day. Fits were performed using constrained optimization to ensure biologically realistic parameter values (*r* ≥ 0, 0 ≤ *y*_∞_ ≤ 100%).

Two distinct fitting windows were used for different analytical purposes: 1. Removal-rate estimation: to characterize initial suppression dynamics, estimates of *r* were derived from fits restricted to the first 6 months following initiation of trapping, corresponding to the longest common early-phase observation window across islands. 2. Equilibrium estimation: to quantify long-term outcomes, estimates of *y*_∞_ were derived from fits to the full available time series for each island, capped at 300 days to ensure comparability while allowing late-phase dynamics to inform equilibrium estimates. This separation avoids biasing removal-rate estimates by late-phase equilibrium dynamics. Optimization was performed using the trust-region reflective algorithm implemented in SciPy (version 1.17).

#### 2.4.3. Uncertainty and Confidence Intervals

Uncertainty in equilibrium abundance estimates was quantified using asymptotic (covariance-based) 95% confidence intervals derived from the variance–covariance matrix of the nonlinear least-squares fit. This approach reflects local curvature of the likelihood surface around the optimum and provides stable uncertainty estimates for well-constrained equilibria.

#### 2.4.4. Trap Density and Comparative Analysis

Trap density was defined as the number of active traps per hectare of island area and was constant within each island throughout the study period. Island-specific equilibrium abundance estimates were compared across the four islands systems as a function of trap density. Because each trap density corresponds to a single island system and trap densities were not experimentally replicated across multiple sites, the relationship between trap density and equilibrium abundance cannot be interpreted as a statistically inferred functional relationship. Instead, the comparison provides an observational assessment of how different trapping intensities correspond to different long-term population outcomes across the four island systems. To aid conceptual interpretation, a decreasing sigmoid-shaped curve is shown as a schematic representation of a potential recruitment–removal transition. No functional relationship was fitted or statistically evaluated between trap density and equilibrium abundance. The curve shown is a schematic illustration only and was not derived from the data. This formulation reflects the theoretical expectation that recruitment–removal systems may exhibit threshold-like dynamics, where increasing removal pressure shifts populations from suppression toward collapse when adult removal exceeds recruitment.

#### 2.4.5. Model Assumptions and AI

The model assumes (i) continuous and constant trap operation, (ii) negligible adult immigration from outside the island system, (iii) homogeneous trapping pressure across each island, and (iv) stable recruitment dynamics over the fitting period. These assumptions are appropriate for island or semi-contained environments and are supported by the sustained trap operation and geographic isolation of the study sites.

ChatGPT (OpenAI, version 5.2) was used to assist with language editing, clarification of scientific phrasing, and coding support for data analysis; all analyses, interpretations, and final text were critically reviewed, verified, and approved by the authors, who take full responsibility for the content of this publication.

## 3. Results

### 3.1. Temporal Dynamics of Aedes Populations Under Sustained Trapping

**Figure 1:**
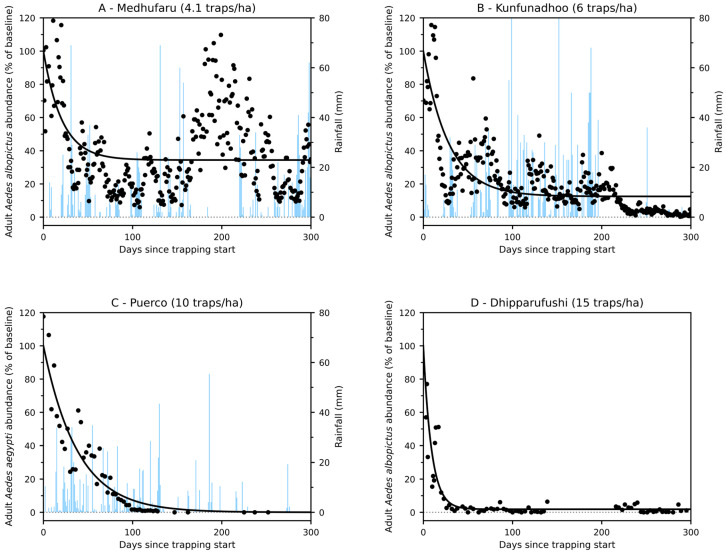
Trap-density–dependent suppression of adult *Ae. albopictus* (**A**,**B**,**D**) or *Ae. aegypti* (**C**) populations across four islands. Time series of adult *Aedes* abundance (points), expressed as a percentage of the pre-intervention baseline (day 0 = 100%), shown for (**A**) Medhufaru (4.1 traps·ha^−1^), (**B**) Kunfunadhoo (6.0 traps·ha^−1^), (**C**) Puerco (10 traps·ha^−1^), and (**D**) Dhipparufushi (15 traps·ha^−1^; there was a gap in data collection between days 139 and 216, but traps were operational during this period). Solid lines represent equilibrium-constrained exponential decay models fitted to the raw data, in which population decline approaches a non-negative long-term equilibrium. All panels use identical axes and display data for up to 300 days following trapping initiation, allowing direct comparison of suppression dynamics across trap densities. Rainfall data (mm/day) are shown as light-blue bars (no data for Dhipparufushi).

Sustained operation of adult BG-Mosquitaire traps resulted in pronounced declines in *Aedes* populations across all four islands, although the magnitude and long-term outcomes differed substantially among sites. When expressed as a percentage of baseline abundance at trap deployment (day 0), all islands exhibited an initial rapid decrease in adult mosquito abundance during the first weeks following the onset of trapping. However, subsequent dynamics differed among islands with different trap densities (Figure 1).

On Medhufaru (4.1 traps·ha^−1^; baseline average 31.7 *Aedes*·trap^−1^·day^−1^ in first two weeks), adult *Aedes albopictus* abundance declined initially but stabilized at a relatively high non-zero level, indicating partial suppression rather than population collapse. The temporary rise in abundance (after day 150) that followed the initial decline was likely caused by a temporary increase in building and construction activities resulting in a sharp increase in potential breeding sites. Kunfunadhoo (approximately 6 traps·ha^−1^; baseline 51.9 *Aedes*·trap^−1^) showed a stronger decline and lower stabilized abundance, yet persistent adult populations remained detectable throughout the observation period. In contrast, on Puerco (10 traps·ha^−1^; baseline density 10.9 *Aedes*·trap^−1^·day^−1^) and Dhipparufushi (15 traps·ha^−1^; baseline density 1.3 *Aedes*·trap^−1^·day^−1^), adult *Aedes* populations declined rapidly and became eliminated, consistent with local population collapse under sustained trapping pressure (we use the term ‘operational elimination’ to denote sustained periods of zero or near-zero adult trap catches, while acknowledging the potential for reintroduction). Puerco island was free of both *Aedes* and *Culex* mosquitoes for 14 months after February 2023 (not a single mosquito was trapped in that period, and no sightings were reported by island inhabitants). Thereafter, the arrival of a building contractor with building materials that contained mosquito eggs (tarpaulins, cement mixers, car tires, etc.) led to the re-establishment of very low-density populations. Across islands, rainfall showed episodic peaks but no consistent relationship with adult abundance. While short-term fluctuations occasionally coincided with rainfall events on Medhufaru and Kunfunadhoo, the overall population trajectories were dominated by sustained declines under trapping. On Puerco, suppression to near-zero levels occurred despite intermittent rainfall. These observations suggest that trapping-driven removal outweighed rainfall effects in shaping long-term population dynamics.

The contrasting outcomes regarding trap densities were robust over time and were not attributable to short-term stochastic fluctuations, as declines on higher-density islands were sustained throughout the monitoring period.

### 3.2. Model Fits and Estimation of Removal and Equilibrium Parameters

The recruitment–removal model provided a good qualitative and quantitative description of observed population trajectories on all islands. Nonlinear least-squares fitting yielded island-specific estimates of the net removal rate (*r*) and long-term equilibrium abundance (*y*_∞_), expressed as a percentage of baseline abundance.

Estimated removal rates varied with trap density, ranging from approximately 0.026 day^−1^ on Puerco to 0.109 day^−1^ on Dhipparufushi (Table 2).

**Figure 2:**
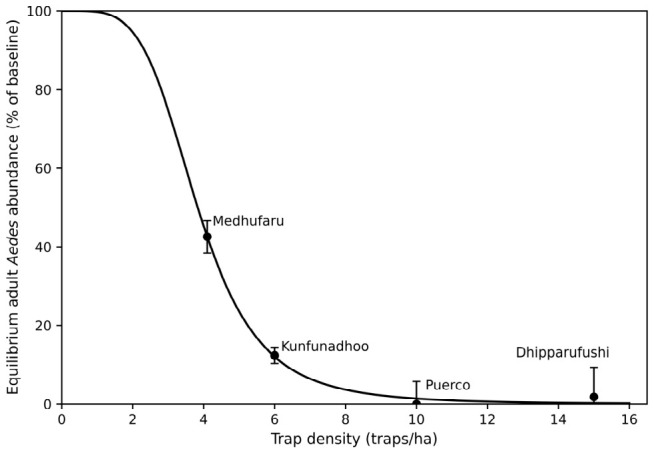
Equilibrium adult *Aedes* abundance (% of baseline) as a function of trap density (traps ha^−1^) across four islands. Equilibrium values correspond to the asymptotic abundance (*y*_∞_) estimated from equilibrium-constrained exponential models fitted to the full available time series for each island, capped at 300 days. Error bars indicate asymptotic (covariance-based) 95% confidence intervals. The decreasing sigmoid curve represents a conceptual illustration of theoretical recruitment–removal dynamics and is shown to illustrate theoretical threshold behavior rather than to provide a statistically fitted model. For Puerco, data refer to *Ae. aegypti*; for the other islands to *Ae. albopictus*.

On Medhufaru, equilibrium abundance stabilized at approximately 43% of baseline, whereas Kunfunadhoo stabilized at approximately 12% of baseline abundance (Table 2 and Figure 2). On Puerco and Dhipparufushi, equilibrium abundance converged toward values indistinguishable from near-zero, consistent with sustained near-zero population levels. At these densities, Puerco reached elimination for 14 months and Dhipparufushi had 2 months with zero trap catches; on both islands re-introduction of eggs occurred thereafter as mentioned before.

Bootstrap resampling indicated relatively larger confidence intervals for equilibrium abundance on islands exhibiting elimination, while smaller intervals were observed on islands with partial suppression, reflecting greater variability in recruitment–removal balance at higher trap densities.

### 3.3. Relationship Between Trap Density and Long-Term Population Outcomes

Across the four island systems, equilibrium adult *Aedes* abundance declined with increasing trap density (Figure 2), with near-zero values observed only at densities ≥ 10 traps ha^−1^. A decreasing exponential curve is shown as a descriptive guide to illustrate this pattern. Given that only four trap densities were available and that each density corresponded to a single island system, this curve serves only as a visual aid and does not represent a fitted model, a statistically supported relationship, or a defined breakpoint.

The comparison across islands is consistent with a transition from partial suppression to near-zero abundance at higher trap densities. However, given that only four systems were available and trap densities were not independently replicated, this pattern should be interpreted as an observational comparison rather than as evidence of a defined functional relationship.

## 4. Discussion

This study provides comparative evidence that sustained mass trapping of adult *Aedes* mosquitoes is associated with fundamentally different population outcomes depending on trap density. Across four tropical island systems, lower equilibrium levels were observed at higher trap densities, with near-zero values occurring only at the highest densities examined. These findings indicate that adult mosquito trapping may function not only as a surveillance or nuisance-reduction tool, but also as a population control intervention capable of overwhelming recruitment when applied at sufficient density.

### 4.1. Threshold Dynamics and Recruitment–Removal Balance

The sigmoid representation is included as a conceptual illustration of how recruitment–removal systems may exhibit threshold-like behavior under increasing removal pressure, rather than as evidence of a defined or estimated critical density. Given the limited number of non-replicated observations, the present dataset does not allow identification of a threshold region or discrimination among alternative functional relationships. Across the four island systems, higher trap densities are associated with lower equilibrium abundances, with near-zero values observed only at the highest densities examined. This pattern is consistent with theoretical expectations, in which increasing adult removal can reduce population size when removal approaches or exceeds local recruitment from immature stages. However, this interpretation does not imply the existence of a discrete breakpoint. Because the study islands differ in size, human population density, vegetation structure, and local mosquito ecology, the comparison between trap density and equilibrium abundance should be interpreted as a pattern across heterogeneous systems rather than as a universally applicable parameter.

Importantly, the transition between suppression and collapse occurred over a relatively narrow range of trap densities, suggesting that modest increases in trap density within the range of densities examined can have disproportionately large effects on population outcomes, a finding with direct operational relevance.

### 4.2. Implications for Vector Control Strategies

Current vector control programs rely heavily on chemical insecticides, particularly space spraying and residual treatments, which face growing challenges due to insecticide resistance, environmental concerns, and non-target impacts. In contrast, mass trapping offers a non-chemical, species-targeted approach that exerts selection pressure primarily through physical removal rather than toxicity. The absence of insecticides reduces risks to biodiversity and may mitigate the rapid evolution of resistance that undermines many chemical interventions.

Compared with emerging biological and genetic approaches—such as *Wolbachia* introgression or gene drive technologies—mass trapping has distinct advantages and limitations. While genetic approaches may achieve self-sustaining population modification or suppression, they require complex regulatory approval, long-term ecological risk assessment, and high levels of public acceptance. Mass trapping, by contrast, is immediately deployable, reversible, and operationally transparent, though it requires sustained infrastructure and maintenance.

The rapid collapse of adult *Aedes* populations observed at trap densities exceeding approximately 10 traps ha^−1^ (Figure 1) suggests that mass trapping may be deployed as a rapid-response vector control measure during outbreaks of *Aedes*-borne diseases. Emergency control guidelines emphasize the importance of interventions that act quickly on the adult vector population, as reductions in adult female abundance can translate into immediate decreases in transmission potential [33,34]. High-density adult trapping targets the epidemiologically relevant life stage and has been associated with substantial population suppression within weeks, contrasting with larval source management or environmental modification, which typically operate on longer timescales. Similar rapid-response logic underpins outbreak interventions such as space spraying and targeted indoor residual spraying, despite their operational and ecological limitations [35,36]. Mass trapping at sufficiently high densities therefore may represent a promising, insecticide-sparing option that could be integrated into outbreak response frameworks, particularly in geographically bounded settings such as islands, urban neighborhoods, or tourism facilities, where rapid deployment, high coverage, and limited reinvasion pressure are achievable.

On Kunfunadhoo and Medhufaru, limited larval source management activities were implemented alongside adult trapping (see Jahir et al. [26] for details), focusing primarily on easily accessible and transient breeding sites. These actions were modest in scale and opportunistic, reflecting the practical constraints of identifying and sustaining comprehensive larval control in complex, vegetated island environments. While such measures may have contributed incrementally to reducing local recruitment, we consider their overall impact to have been secondary to that of adult mass trapping. Notably, on Puerco no larval source management was undertaken, yet elimination was nevertheless observed [27]. This contrast strengthens the inference that sustained removal of adult females, rather than reductions in larval productivity, was the dominant driver of suppression. At the same time, integrated vector management (combining adult control, larval source management, environmental modification, and community engagement) remains the recommended framework for sustainable vector control across diverse epidemiological and ecological settings beyond small, geographically bounded islands [33,37].

### 4.3. Species Considerations and Generality of Findings

These findings apply to *Ae. aegypti* and *Ae. albopictus* under the specific trapping conditions used here. Extrapolation to other *Aedes* species, particularly those with different host-seeking behaviors and/or host range, requires further study. Species-specific behavioral differences and trapping efficiency for different *Aedes* species may influence long-term equilibria. *Aedes albopictus*, for example, may exhibit different host-seeking and dispersal behaviors that affect trap encounter rates compared with *Ae. aegypti*. Dynamics may further be affected in areas where both species occur sympatrically. Further comparative studies across species and ecological contexts are warranted to refine the potential impacts of mass trapping on mosquito populations.

### 4.4. Operational and Spatial Considerations

The island setting was critical to the success of the trapping interventions studied here. Geographic isolation limited adult immigration, allowing local recruitment dynamics to dominate population outcomes. In more open or urban environments, immigration from surrounding areas may substantially increase the trap density required to achieve comparable effects. Nonetheless, many real-world settings (such as islands, resorts, gated communities, industrial sites, and peri-urban zones) share sufficient spatial containment to make threshold-based trapping strategies feasible.

The assumption of homogeneous trap density may also mask fine-scale spatial effects. Clustering of breeding sites or uneven human activity could locally reduce trapping efficiency, suggesting that adaptive placement strategies may further improve outcomes without necessarily increasing total trap numbers.

### 4.5. Policy and Research Implications

These findings provide a quantitative framework for designing mass trapping programs based on explicit population-level objectives rather than empirical trial-and-error. Determining appropriate trap densities for specific operational settings can guide resource allocation, cost-effectiveness analyses, and integration with other interventions such as larval source management.

Future research should expand the number of study sites, explore intermediate trap densities, and incorporate mechanistic life-stage models to explicitly link adult removal with larval dynamics and environmental drivers. Integrating mass trapping into broader integrated vector management strategies may offer a robust and environmentally compatible pathway for controlling *Aedes*-borne diseases in appropriate settings.

### 4.6. Limitations

Several limitations should be considered when interpreting the results of this study. First, the population model employed is intentionally simple and phenomenological. While the recruitment–removal formulation captures the dominant dynamics observed under sustained mass trapping, it does not explicitly represent mosquito life stages, density-dependent larval competition, or weather-driven variation in recruitment. As a result, short-term fluctuations linked to rainfall or episodic breeding events are not mechanistically modeled but are instead absorbed into the equilibrium parameter.

Second, the analysis assumes negligible adult mosquito immigration from outside the study areas. Although this assumption is well supported for island and semi-contained systems, it may not hold on the mainland or in highly connected urban environments. Consequently, the range of trap densities examined here should be interpreted as applicable primarily to isolated or semi-isolated settings, and extrapolation to open systems should be undertaken with caution.

Third, trap density was treated as spatially homogeneous within each island. In practice, micro-scale variation in trap placement, human activity, vegetation structure, and breeding site distribution may lead to localized heterogeneity in trapping pressure. Such spatial effects were not explicitly modeled and could influence fine-scale population dynamics.

Fourth, species-specific behavioral and ecological differences were not explicitly incorporated into the model structure. Although the study included both *Aedes aegypti* and *Ae. albopictus*, the same modeling framework was applied to all islands. Differences in host-seeking behavior (i.e., the level of anthropophily), dispersal, and breeding ecology between species may affect trapping efficiency and recruitment rates, potentially influencing comparisons across trap densities.

Finally, the number of islands included in the threshold analysis was limited. While the four study systems spanned a broad range of trap densities and yielded consistent patterns, additional data from intermediate densities and diverse ecological contexts would further refine understanding of how outcomes vary across trap densities and improve generalizability.

Despite these limitations, the consistency of results across independent island systems indicates a clear directional pattern in population outcomes across the range of trap densities examined.

## 5. Conclusions

This study provides comparative evidence that sustained mass trapping of adult *Aedes* mosquitoes can produce markedly different population outcomes depending on trap density. Using comparative data from four tropical island systems, we describe that low to moderate trap densities lead to partial suppression, whereas higher trap densities consistently drive mosquito populations toward near-zero equilibrium levels. Although these patterns are consistent across the systems examined, the limited number of trap densities and lack of independent replication preclude formal identification of a functional relationship. Thus, further studies across a wider range of trap densities are required to evaluate the shape of this relationship.

Our findings provide comparative evidence that adult mosquito trapping, when implemented at sufficient density and maintained continuously, may function as a population control strategy rather than solely as a surveillance or nuisance-reduction tool. Moreover, the observed differences across trap densities offers a practical basis for designing and evaluating mass trapping programs based on defined population-level objectives.

Mass trapping represents a non-chemical, reversible, and environmentally compatible approach that may complement existing vector control strategies, particularly in geographically isolated or semi-contained settings where mosquito immigration is limited. While extrapolation to open systems requires caution, the consistency of outcomes across islands differing in size, human occupancy, and *Aedes* species suggests that strong suppression at higher trap densities may be achievable.

Together, these results support consideration of the integration of high-density adult trapping into integrated vector management programs and highlight the value of quantitative, population-based approaches for optimizing mosquito control interventions.

## Figures and Tables

**Table 1:** Study islands (M = Maldives; P = Philippines) with central coordinates, size, human population, trap density and *Aedes* species trapped/monitored.

Island	Latitude (°N, WGS84)	Longitude (°E, WGS84)	Area (ha)	Trap Density (Traps·ha^−1^)	Inhabitants (approx.)	*Aedes* Species
Medhufaru (M)	5.71333	73.41472	49.0	4.1	500–700	*Ae. albopictus*
Kunfunadhoo (M)	5.11222	73.07722	41.4	6.0	420–700	*Ae. albopictus*
Puerco (P)	10.31861	119.48194	7.2	10.0	70–100	*Ae. aegypti*
Dhipparufushi (M)	6.31889	72.64056	3.0	15.0	150–200	*Ae. albopictus*

Central coordinates represent the geographic centroid of each island (WGS84). Island area corresponds to the operational study area used for trap-density calculations. Population estimates reflect typical occupancy during the study period but witness short-term increases of 50–70% during holiday periods.

**Table 2:** Baseline adult *Aedes* abundance on four islands, deployed trap densities, and the resulting removal rate (*r*) and equilibrium abundance (as % of baseline). Baseline abundance reflects *Aedes* catches per day per trap over the first 5 sampling events (~2 weeks); removal rate was calculated over the first 6 months, and equilibrium abundance over 300 days.

Island	Target Species	Baseline Abundance (*y*_0_)	Trap Density (Traps·ha^−1^)	Removal Rate *r* (day^−1^) [95% CI]	Equilibrium *y*_∞_ (% of Baseline) [95% CI]
Medhufaru	*Ae. albopictus*	31.7	4.1	0.029 [0.022–0.061]	42.6 [38.4–46.7]
Kunfunadhoo	*Ae. albopictus*	59.1	6.0	0.037 [0.031–0.042]	12.4 [10.4–14.4]
Puerco	*Ae. aegypti*	10.9	10.0	0.026 [0.019–0.033]	~0.0 [0.0–5.8]
Dhipparufushi	*Ae. albopictus*	1.3	15.0	0.109 [0.047–0.171]	1.8 [0.0–9.4]

## Data Availability

All raw trap data for the four islands used in the analysis are available as Supplementary Materials.

## Supplementary Materials

The following supporting information can be downloaded at: https://www.mdpi.com/article/10.3390/insects17050472/s1.

## References

[1] Bhatt S., Gething P.W., Brady O.J., Messina J.P., Farlow A.W., Moyes C.L., Drake J.M., Brownstein J.S., Hoen A.G., Sankoh O. (2013). The global distribution and burden of dengue. Nature.

[2] World Health Organization (2024). Dengue and Severe Dengue; WHO Fact Sheet.

[3] World Health Organization (2025). Dengue: Global Situation, Surveillance and Progress—2024 Update.

[4] Brady O.J., Golding N., Pigott D.M., Kraemer M.U., Messina J.P., Reiner R.C., Scott T.W., Smith D.L., Gething P.W., Hay S.I. (2014). Global temperature constraints on *Aedes aegypti* and *Ae. albopictus* persistence and competence for dengue virus transmission. Parasit. Vectors.

[5] Hedrich N., Bekker-Nielsen Dunbar M., Grobusch M.P., Schlagenhauf P. (2025). Aedes-borne arboviral human infections in Europe from 2000 to 2023: A systematic review and meta-analysis. Travel Med. Infect. Dis..

[6] Radici A., Hammami P., Cannet A., L’Ambert G., Lacour G., Fournet F., Garros C., Guis H., Fontenille D., Caminade C. (2025). *Aedes albopictus* Is Rapidly Invading Its Climatic Niche in France: Wider Implications for Biting Nuisance and Arbovirus Control in Western Europe. Glob. Change Biol..

[7] Willoughby J.R., McKenzie B.A., Ahn J., Steury T.D., Lepzcyk C.A., Zohdy S. (2024). Assessing and managing the risk of *Aedes* mosquito introductions via the global maritime trade network. PLoS Negl. Trop. Dis..

[8] Deblauwe I., De Wolf K., De Witte J., Schneider A., Verlé I., Vanslembrouck A., Smitz N., Demeulemeester J., Van Loo T., Dekoninck W. (2022). From a long-distance threat to the invasion front: A review of the invasive *Aedes* mosquito species in Belgium between 2007 and 2020. Parasit. Vectors.

[9] Wilder-Smith A., Gubler D.J., Weaver S.C., Monath T.P., Heymann D.L., Scott T.W. (2017). Epidemic arboviral diseases: Priorities for research and public health. Lancet Infect. Dis..

[10] Ryan S.J., Carlson C.J., Mordecai E.A., Johnson L.R. (2019). Global expansion and redistribution of *Aedes*-borne virus transmission risk with climate change. PLoS Negl. Trop. Dis..

[11] Caminade C., McIntyre K.M., Jones A.E. (2019). Impact of recent and future climate change on vector-borne diseases. Ann. N. Y. Acad. Sci..

[12] Mordecai E.A., Caldwell J.M., Grossman M.K., Lippi C.A., Johnson L.R., Neira M., Rohr J.R., Ryan S.J., Savage V., Shocket M.S. (2019). Thermal biology of mosquito-borne disease. Ecol. Lett..

[13] Gubler D.J. (2011). Dengue, urbanization and globalization: The unholy trinity of the 21st century. Trop. Med. Health.

[14] Dusfour I., Vontas J., David J.P., Weetman D., Fonseca D.M., Corbel V., Raghavendra K., Coulibaly M.B., Martins A.J., Kasai S. (2019). Management of insecticide resistance in the major *Aedes* vectors of arboviruses: Advances and challenges. PLoS Negl. Trop. Dis..

[15] Moyes C.L., Vontas J., Martins A.J., Ng L.C., Koou S.Y., Dusfour I., Raghavendra K., Pinto J., Corbel V., David J.P. (2017). Contemporary status of insecticide resistance in the major *Aedes* vectors of arboviruses infecting humans. PLoS Negl. Trop. Dis..

[16] Sánchez-Bayo F., Wyckhuys K.A. (2019). Worldwide decline of the entomofauna: A review of its drivers. Biol. Conserv..

[17] Lee N.S.M., Clements G.R., Ting A.S.Y., Wong Z.H., Yek S.H. (2020). Persistent mosquito fogging can be detrimental to non-target invertebrates in an urban tropical forest. PeerJ.

[18] Milman O. (2022). The Insect Crisis: The Fall of the Tiny Empires That Run the World.

[19] O’Neill S.L., Ryan P.A., Turley A.P., Wilson G., Retzki K., Iturbe-Ormaetxe I., Dong Y., Kenny N., Paton C.J., Ritchie S.A. (2018). Scaled deployment of *Wolbachia* to protect the community from dengue and other *Aedes*-transmitted arboviruses. Gates Open Res..

[20] Lees R.S., Gilles J.R.L., Hendrichs J., Vreysen M.J.B., Bourtzis K. (2015). Back to the future: The sterile insect technique against mosquito disease vectors. Curr. Opin. Insect Sci..

[21] Burt A. (2014). Heritable strategies for controlling insect vectors of disease. Phil. Trans. R. Soc. B.

[22] Li M., Kandul N.P., Sun R., Yang T., Benetta E.D., Brogan D.J., Antoshechkin I., Sánchez C.H.M., Zhan Y., DeBeaubien N.A. (2024). Targeting sex determination to suppress mosquito populations. Elife.

[23] Jaffal A., Fite J., Baldet T., Delaunay P., Jourdain F., Mora-Castillo R., Olive M.M., Roiz D. (2023). Current evidences of the efficacy of mosquito mass-trapping interventions to reduce *Aedes aegypti* and *Aedes albopictus* populations and *Aedes*-borne virus transmission. PLoS Negl. Trop. Dis..

[24] Degener C.M., Geier M., Kline D., Urban J., Willis S., Ramirez K., Cloherty E.R., Gordon S.W. (2019). Field trials to evaluate the effectiveness of the Biogents^®^-Sweetscent lure in combination with several commercial mosquito traps and to assess the effectiveness of the Biogents-Mosquitaire trap with and without carbon dioxide. J. Am. Mosq. Control Assoc..

[25] Degener C.M., Staunton K.M., Bossin H., Marie J., da Silva R.D., Lima D.C., Eiras Á.E., Akaratovic K.I., Kiser J., Gordon S.W. (2021). Evaluation of the new modular Biogents BG-Pro mosquito trap in comparison to CDC, EVS, BG-Sentinel, and BG-Mosquitaire traps. J. Am. Mosq. Control Assoc..

[26] Jahir A., Kahamba N.F., Knols T.O., Jackson G., Patty N.F.A., Shivdasani S., Okumu F.O., Knols B.G. (2022). Mass Trapping and Larval Source Management for Mosquito Elimination on Small Maldivian Islands. Insects.

[27] Knols B.G.J., Posada A., Sison M.J., Knols J.M.H., Patty N.F.A., Jahir A. (2023). Rapid Elimination of *Aedes aegypti* and *Culex quinquefasciatus* Mosquitoes from Puerco Island, Palawan, Philippines with Odor-Baited Traps. Insects.

[28] Barclay H., van den Driessche P. (1983). Pheromone trapping models for insect pest control. Pop. Ecol..

[29] Anguelov R., Dufourd C., Dumont Y. (2017). Mathematical model for pest-insect control using mating disruption and trapping. Appl. Math. Model..

[30] Barclay H., Mackauer M. (1980). The Sterile Insect Release Method for Pest Control: A Density-Dependent Model. Environ. Entomol..

[31] Johnson B.J., Ritchie S.A., Fonseca D.M. (2017). The State of the Art of Lethal Oviposition Trap-Based Mass Interventions for Arboviral Control. Insects.

[32] Service M.W. (1993). Mosquito Ecology: Field Sampling Methods.

[33] World Health Organization (2017). Global Vector Control Response 2017–2030.

[34] World Health Organization (2023). Guidelines for Dengue Surveillance and Vector Control.

[35] Achee N.L., Gould F., Perkins T.A., Reiner R.C., Morrison A.C., Ritchie S.A., Gubler D.J., Teyssou R., Scott T.W. (2015). A critical assessment of vector control for dengue prevention. PLoS Negl. Trop. Dis..

[36] Bowman L.R., Donegan S., McCall P.J. (2016). Is dengue vector control deficient in effectiveness or evidence?. PLoS Negl. Trop. Dis..

[37] World Health Organization (2012). Handbook for Integrated Vector Management.

